# Rural Telemedicine Use Before and During the COVID-19 Pandemic: Repeated Cross-sectional Study

**DOI:** 10.2196/26960

**Published:** 2021-04-05

**Authors:** Cherry Chu, Peter Cram, Andrea Pang, Vess Stamenova, Mina Tadrous, R Sacha Bhatia

**Affiliations:** 1 Women's College Hospital Institute for Health System Solutions and Virtual Care Women's College Hospital Toronto, ON Canada; 2 Division of General Internal Medicine and Geriatrics Sinai Health System and University Health Network Toronto, ON Canada; 3 Department of Medicine University of Toronto Toronto, ON Canada; 4 Institute for Clinical Evaluative Sciences Toronto, ON Canada; 5 Leslie Dan Faculty of Pharmacy University of Toronto Toronto, ON Canada

**Keywords:** chronic disease, chronic illness, COVID-19, health care, health services, older adults, remote, rural, pandemic, population, telemedicine, virtual care

## Abstract

**Background:**

The COVID-19 pandemic has led to a notable increase in telemedicine adoption. However, the impact of the pandemic on telemedicine use at a population level in rural and remote settings remains unclear.

**Objective:**

This study aimed to evaluate changes in the rate of telemedicine use among rural populations and identify patient characteristics associated with telemedicine use prior to and during the pandemic.

**Methods:**

We conducted a repeated cross-sectional study on all monthly and quarterly rural telemedicine visits from January 2012 to June 2020, using administrative data from Ontario, Canada. We compared the changes in telemedicine use among residents of rural and urban regions of Ontario prior to and during the pandemic.

**Results:**

Before the pandemic, telemedicine use was steadily low in 2012-2019 for both rural and urban populations but slightly higher overall for rural patients (11 visits per 1000 patients vs 7 visits per 1000 patients in December 2019, *P*<.001). The rate of telemedicine visits among rural patients significantly increased to 147 visits per 1000 patients in June 2020. A similar but steeper increase (*P*=.15) was observed among urban patients (220 visits per 1000 urban patients). Telemedicine use increased across all age groups, with the highest rates reported among older adults aged ≥65 years (77 visits per 100 patients in 2020). The proportions of patients with at least 1 telemedicine visit were similar across the adult age groups (n=82,246/290,401, 28.3% for patients aged 18-49 years, n=79,339/290,401, 27.3% for patients aged 50-64 years, and n=80,833/290,401, 27.8% for patients aged 65-79 years), but lower among younger patients <18 years (n=23,699/290,401, 8.2%) and older patients ≥80 years (n=24,284/290,401, 8.4%) in 2020 (*P*<.001). There were more female users than male users of telemedicine (n=158,643/290,401, 54.6% vs n=131,758/290,401, 45.4%, respectively, in 2020; *P*<.001). There was a significantly higher proportion of telemedicine users residing in relatively less rural than in more rural regions (n=261,814/290,401, 90.2% vs n=28,587/290,401, 9.8%, respectively, in 2020; *P*<.001).

**Conclusions:**

Telemedicine adoption increased in rural and remote areas during the COVID-19 pandemic, but its use increased in urban and less rural populations. Future studies should investigate the potential barriers to telemedicine use among rural patients and the impact of rural telemedicine on patient health care utilization and outcomes.

## Introduction

Access to health care services in rural communities has proven challenging among individuals for many reasons. There is a scarcity of health care professionals serving rural populations, and consequently, rural residents face barriers to health care access, such as long travel times, limited public transportation and associated costs, and time away from work. Ontario is Canada’s most populous province with over 14,000,000 people, and, at >1,000,000 km^2^, it has a larger area than Texas and Montana combined. While most of Ontario’s population is situated in the southern region of the province in urban areas, approximately 10% of the population resides in rural areas [1], including the northern regions of the province. The Ontario Telemedicine Network (OTN) is an organization funded by the provincial government, which aimed to initially increase health care access among the rural population by facilitating 2-way videoconferencing between patients and care providers [2]. However, prior to the COVID-19 pandemic, telemedicine adoption was modest in Ontario [3], in part owing to strict requirements for both the patient and the physician, such as attending a virtual visit at an OTN facility, and using an OTN-approved communication platform [4]. A prepandemic study from Ontario revealed that Northern Ontario, a predominantly rural region in the province, saw markedly higher annual rates of telemedicine use than the mostly urban Southern Ontario, and even within Northern Ontario, rates of telemedicine use were higher in the “very rural” than in the “less rural” populations [5].

The COVID-19 pandemic has posed a unique challenge for rural patients—particularly those who are more susceptible to the disease—such as older adults and those with chronic diseases. In particular, there has been a need to balance chronic disease management with the risk of virus transmission, either during health care consultations or when traveling from rural to urban areas [6]. In order to reduce virus transmission in the health care setting, the Ontario Health Insurance Plan (OHIP)—the government health insurer across the province—introduced temporary billing codes that broadened the range of modalities eligible for reimbursement beyond OTN-approved videoconferencing platforms, including telephone calls and many other types of technology [7]. While a limited number of studies have reported substantial increases in the use of telemedicine during the pandemic, there are currently no published data on the use of telemedicine in rural communities or on the potential differences in telemedicine uptake between rural and urban populations during the pandemic. An understanding of rural telemedicine uptake and how it has been affected by the COVID-19 pandemic can provide insight into the utility of telemedicine in rural areas, particularly among vulnerable and at-risk populations; moreover, this information can contribute to the development of targeted approaches to increase telemedicine access where uptake was limited.

This study aimed to measure the utilization of telemedicine in rural and urban populations and among at-risk patient groups in rural Ontario prior to and during the COVID-19 pandemic, and to describe the characteristics of rural patients who have used telemedicine.

## Methods

### Study Design and Data Sources

We conducted a population-based, repeated cross-sectional study of monthly and quarterly rural ambulatory telemedicine visits in Ontario, Canada, beginning prior to the COVID-19 pandemic (January 1, 2012) and extending to June 30, 2020, using data from the following administrative databases: (1) OHIP, which records all health services delivered by physicians to Ontario residents and (2) Registered Persons Database, which contains demographic information of all patients covered under OHIP. We linked each patient’s postal code of residence to census-based neighborhood income measures and then stratified patients across the province into neighborhood income quintiles. To understand telemedicine uptake among patients with various chronic conditions, we defined individual patient cohorts on the basis of select chronic ambulatory care conditions, using the Discharge Abstract Database, which records all in-patient hospital admissions, the National Ambulatory Care Reporting System, which contains data on all hospital- and community-based ambulatory care (including emergency department visits), and various Institute for Clinical Evaluative Sciences (ICES)–validated disease-specific registries. ICES is an independent, nonprofit research institute whose legal status under Ontario’s health information privacy law allows it to collect and analyze health care and demographic data without consent for health system evaluation and improvement. Databases were linked using unique encoded identifiers and analyzed at the ICES. The use of these databases for the purpose of this study was authorized under clause 45 of Ontario’s Personal Health Information Protection Act [8], which does not require review by a research ethics board.

### Population

We identified all patients residing in the rural regions of Ontario by using the rurality index for Ontario (RIO) and their ambulatory visits through telemedicine through relevant physician billing codes (Multimedia Appendix 1). The RIO score has been established by the Ministry of Health and Long-Term Care as a method to fairly and consistently measure a community’s degree of rurality based on its postal code in order to allocate governmental funding for rural health programs. The RIO score is derived from three measures: population density, distance from a basic referral center, and distance from an advanced referral center [9]. A higher RIO score indicates a higher degree of rurality, with a maximum score of 100. Rural residents have been defined as patients with a RIO score of ≥40, which is a standard cut-off used by the provincial government [10]. We compared the rates of rural telemedicine visits to those of urban visits, the latter defined with a RIO score of <40. We excluded claims for any patient who was a nonresident of Ontario or had an invalid or missing health card number.

We further identified patients diagnosed with chronic disease including chronic obstructive pulmonary disease, congestive heart failure, asthma, hypertension, and diabetes mellitus from the existing established ICES registries and algorithms [11]. Patients with serious mental illness were identified from at least 2 outpatient or 1 inpatient claims with the corresponding International Classification of Diseases, 9th revision or 10th revision codes for schizophrenia, psychotic disorders, or bipolar disorder in the past 12 months. Patients with angina were identified by at least one emergency department visit with the aforementioned relevant disease codes in the past 12 months (Multimedia Appendix 1).

### Statistical Analysis

For each month in our study period (January 1, 2012, to June 30, 2020), we reported the rate of telemedicine visits per 1000 patients in rural Ontario. Given the markedly high population of older adults in rural Ontario [12], we also calculated quarterly rates separately among patients aged ≥65 years and compared them with those of other age groups (visits per 100 patients). Chi-square tests were conducted to assess the distribution of various characteristics of patients who have used telemedicine (age category, sex, region of residence, neighborhood income quintile, level of rurality, and chronic disease diagnosis) across 2012, 2016, and 2020. We selected 2012 as the first year of the study period, 2016 as the middle period, and 2020 as the pandemic period. We also compared quarterly telemedicine utilization across chronic disease subgroups (visits per 100 patients). A *P* value less than .05 was considered significant. All analyses were performed using SAS (version 9.4, The SAS Institute).

## Results

We compared the characteristics of rural patients who used telemedicine across the calendar years of 2012 and 2016 and the first half of 2020. The number of rural patients who received at least one telemedicine visit increased from 2012 (n=14,666/1,017,546, 1.4%) to 2020 (n=290,401/1,033,271, 28.1%). Prior to the pandemic, a greater proportion of adults aged 18-49 years used telemedicine (n=4965/14,666, 33.9% in 2012) compared to the other age groups (n=4140/14,666, 28.2% for patients aged 50-64 years and n=3716/14,666, 25.3% for patients aged 65-79 years), but after the onset of the pandemic, we observed similar proportions of telemedicine users among the older age groups as well (n=82,246/290,401, 28.3% for patients aged 18-49 years; n=79,339/290,401, 27.3% for patients aged 50-64 years, and n=80,833/290,401, 27.8% for patients aged 65-79 years). In 2012 and 2016, there was an approximately equal proportion of male and female telemedicine users; however, in 2020, there were more female users than male users (n=158,643/290,401, 54.6% vs n=131,758, 45.4%, respectively). Prior to the COVID-19 pandemic, most users were based in Northern Ontario (n=10,431/14,666, 71.1% in 2012 and n=15,702/27,145, 57.8% in 2016), but after the onset of the pandemic, there was a more balanced distribution of users across all regions, with the highest proportion of users in Eastern Ontario (n=98,008/290,401, 33.7% in 2020). These results are summarized in Table 1. Similarly, in 2012, the highest rate of telemedicine visits was observed among patients residing in Northern Ontario; however, the Central and Eastern regions recorded the highest rates in 2020 (Multimedia Appendix 2). Smaller shifts were reported in telemedicine use across income quintiles, with more patients in the lower-income quintiles using telemedicine in 2012 (n=3270/14,666, 22.3% for quintile 1 and n=3013/14,666, 20.5% for quintile 2) and 2016 (n=6012/27,145, 22.1% for quintile 1 and n=6770/27,145, 24.9% for quintile 2) but more users in the middle-income quintiles in 2020 (n=67,172/290,401, 23.1% in quintile 2 and n=65,128/290,401, 22.4% in quintile 3). In 2012, we observed a higher proportion of telemedicine users living in less rural areas (lower RIO score) than in more rural areas (n=8709/14,666, 59.4% vs n=5957/14,666, 40.6%, respectively). This difference further increased across the years, with the largest difference observed in 2020 (n=261,814/290,401, 90.2% vs n=28,587/290,401, 9.8%, respectively). While the absolute number of telemedicine users with chronic disease has increased significantly over the years, for most chronic disease groups, the proportion of users with the disease appeared to decrease slightly (eg, 1041 of 14,666 [7.1%] patients had heart failure in 2012 as opposed to 12,580 of 290,401 [4.3%] in 2020; *P*<.001). Among the health conditions of interest in this study, hypertension was the most frequently diagnosed condition (n=112,297/290,401, 38.7% in 2020), followed by diabetes (n=52,830/290,401, 18.2% in 2020).

**Figure 1:**
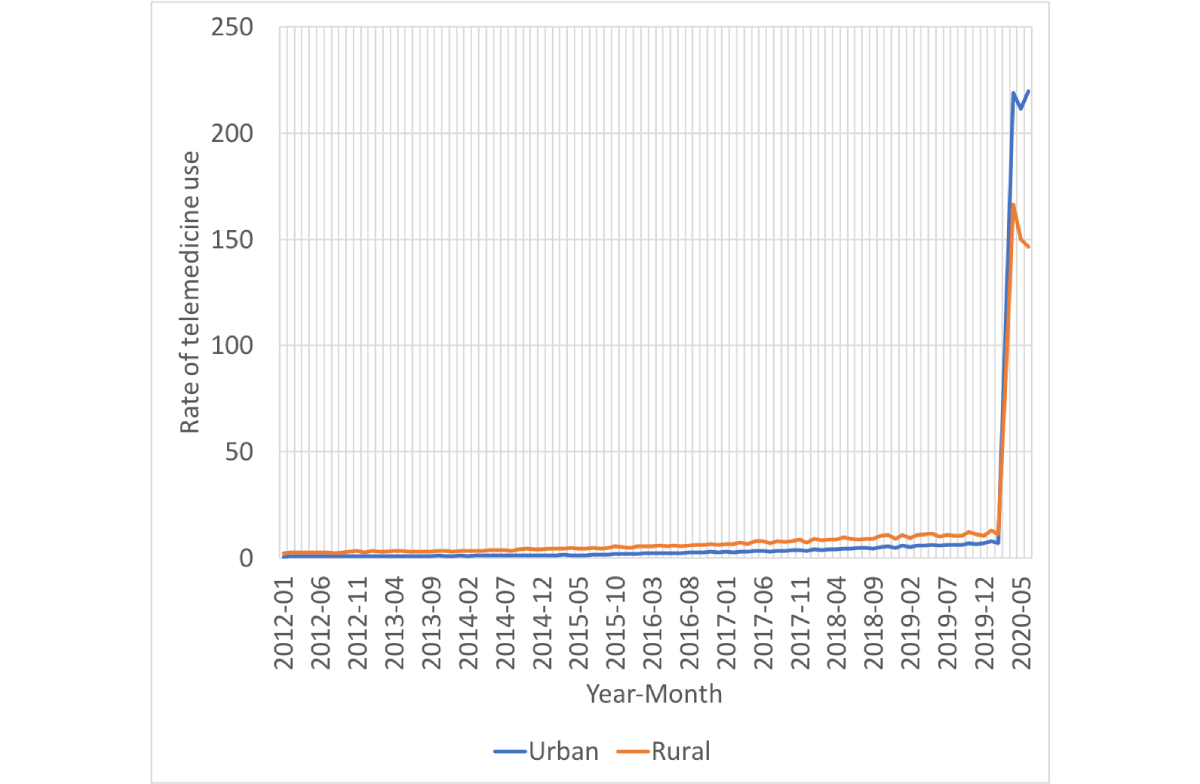
Rate of telemedicine visits per 1000 eligible patients in Ontario by rurality, 2012-2020.

We observed a significant increase in the rate of telemedicine visits among both rural and urban patients after the start of the COVID-19 pandemic. Prior to the pandemic, this rate was consistently higher among rural patients than among urban patients (11 visits per 1000 patients vs 7 visits per 1000 patients in December 2019, respectively; *P*<.001). During the pandemic, both urban and rural telemedicine usage rates increased significantly, but utilization rates among rural patients were lower than those among urban patients (147 visits per 1000 patients vs 220 visits per 1000 patients in June 2020, respectively; *P*=.15). These findings are illustrated in Figure 1.

Among physicians who provided telemedicine consultations to rural patients, there was a large shift from mostly in-person visits in 2012 and 2016 to telemedicine visits in 2020. The highest proportion of telemedicine providers in 2020 were from family or general practice (n=9044/17,601, 51.4%) (Multimedia Appendix 3).

**Figure 2:**
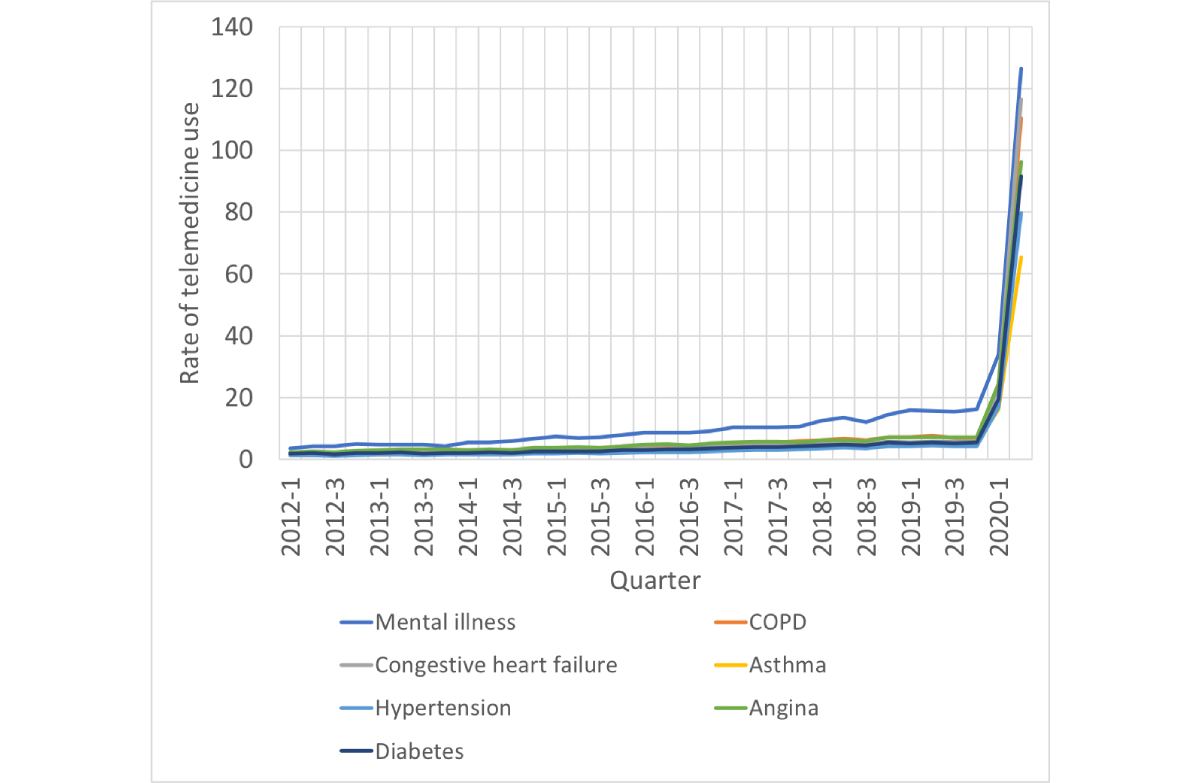
Rate of telemedicine visits per 100 rural patients by chronic disease, 2012-2020. COPD: chronic obstructive pulmonary disease.

The rate of telemedicine visits increased significantly from before to during the COVID-19 pandemic among patients with various chronic disease conditions, with the highest rates observed among patients in the mental illness subgroup (126 visits per 100 patients in in second quarter of 2020), followed by those in the congestive heart failure (116 visits per 100 patients), chronic obstructive pulmonary disease (110 visits per 100 patients), angina (96 visits per 100 patients), diabetes (92 visits per 100 patients), hypertension (80 visits per 100 patients), and asthma (65 visits per 100 patients) subgroups. These trends are shown in Figure 2.

**Figure 3:**
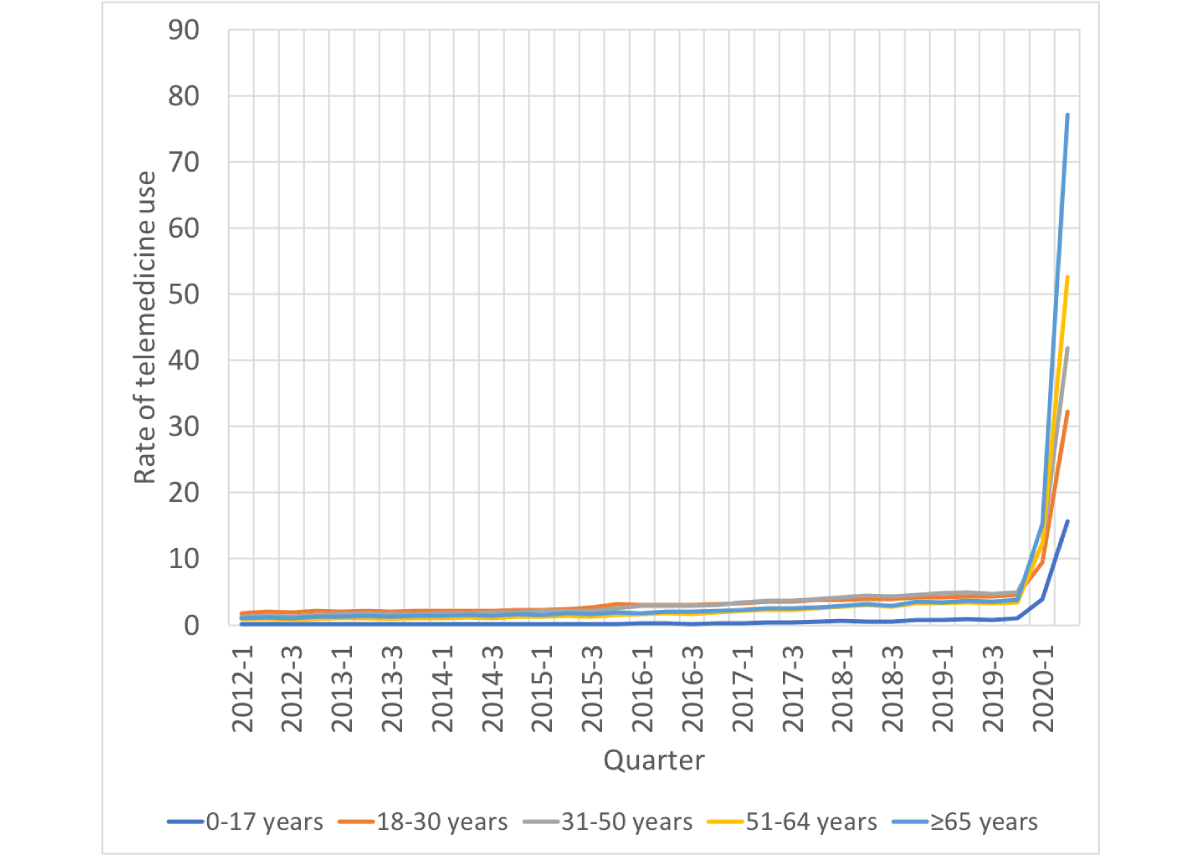
Rate of telemedicine visits per 100 rural patients by age group, 2012-2020.

Prior to the pandemic, the rates of telemedicine visits were low and stable; however, the onset of the pandemic led to a surge in telemedicine use across all age groups. The highest rates were reported among adults aged ≥65 years, approaching 77 visits per 100 patients in the second quarter of 2020 as opposed to 4 visits per 100 patients in the fourth quarter of 2019. The next highest telemedicine usage rates during the pandemic was observed among patients aged 51-64 years (53 visits per 100 patients), followed by those aged 31-50 years (42 visits per 100 patients), and those aged 18-30 years (32 visits per 100 patients). The lowest rates of telemedicine use were observed among the youngest group of patients (aged 0-17 years, 16 visits per 100 patients). These findings are illustrated in Figure 3.

**Table 1:** Characteristics of rural patients who received at least 1 telemedicine visit in 2012, 2016, and 2020.

Variables		Number of patients, n (%)			*P* value	
		2012 (n=14,666)	2016 (n=27,145)	2020 (n=290,401)		
**Age group (years)**						<.001
	<18	565 (3.9)	1011 (3.7)	23,699 (8.2)		
	18-49	4965 (33.9)	9146 (33.7)	82,246 (28.3)		
	50-64	4140 (28.2)	7917 (29.2)	79,339 (27.3)		
	65-79	3716 (25.3)	6841 (25.2)	80,833 (27.8)		
	≥80	1280 (8.7)	2230 (8.2)	24,284 (8.4)		
**Sex**						<.001
	Female	7345 (50.1)	13,343 (49.2)	158,643 (54.6)		
	Male	7321 (49.9)	13,802 (50.8)	131,758 (45.4)		
**Region of Ontario**						<.001
	Central	775 (5.3)	1870 (6.9)	48,526 (16.7)		
	East	2886 (19.7)	7042 (25.9)	98,008 (33.7)		
	North	10,431 (71.1)	15,702 (57.8)	69,552 (24.0)		
	West	574 (3.9)	2531 (9.3)	74,315 (25.6)		
**Income quintile**						<.001
	1 (lowest)	3270 (22.3)	6012 (22.1)	53,448 (18.4)		
	2	3013 (20.5)	6770 (24.9)	67,172 (23.1)		
	3	2868 (19.6)	5698 (21.0)	65,128 (22.4)		
	4	2482 (16.9)	4713 (17.4)	59,767 (20.6)		
	5 (Highest)	2965 (20.2)	3952 (14.6)	44,885 (15.5)		
**Level of rurality (rurality index of Ontario score)**						<.001
	40-75 (less rural)	8709 (59.4)	19,212 (70.8)	261,814 (90.2)		
	76-100 (more rural)	5957 (40.6)	7933 (29.2)	28,587 (9.8)		
**Chronic disease**						
	Hypertension	6239 (42.5)	11,300 (41.6)	112,297 (38.7)	<.001	
	Diabetes	3451 (23.5)	5920 (21.8)	52,830 (18.2)	<.001	
	Chronic obstructive pulmonary disease	1371 (9.3)	2600 (9.6)	16,941 (5.8)	<.001	
	Congestive heart failure	1041 (7.1)	1957 (7.2)	12,580 (4.3)	<.001	
	Asthma	1584 (10.8)	3328 (12.3)	33,223 (11.4)	<.001	
	Angina	679 (4.6)	1239 (4.6)	8720 (3.0)	<.001	
	Mental illness	505 (3.4)	1011 (3.7)	4855 (1.7)	<.001	

## Discussion

### Principal Findings

In this population-based study, we sought to quantify the impact of the COVID-19 pandemic on rural telemedicine use across a large, geographically and demographically diverse region. While telemedicine use before the COVID-19 pandemic was higher in rural areas than in urban areas of Ontario, the use of telemedicine was modest. Broad telemedicine usage in Ontario was low in part owing to the lack of billing codes for many types of telemedicine services outside of the government-operated videoconferencing platform. With the initiation of these temporary fee-codes allowing reimbursement for other forms of telemedicine visits during the pandemic, usage increased rapidly, with the temporary visit codes comprising the vast majority of telemedicine visits. The onset of the pandemic led to a rapid increase in telemedicine usage in both rural and urban areas of Ontario, with high adoption of telemedicine among patients in urban areas and those in less rural areas, compared to those in the most remote regions of Ontario. We speculate that higher rates of urban telemedicine use during the pandemic coincide with generally higher rates of urban health care use as telemedicine became ubiquitous. We note that the slight reduction or plateau in the telemedicine usage rates among both urban and rural populations from April to June 2020 coincided with the loosening of lockdown restrictions after the first wave of the COVID-19 pandemic and may have resulted from an increase in in-person visits during this period.

Telemedicine usage increased rapidly among all age groups in rural areas, although the usage rate increased with age. Older adult patients used telemedicine at the highest rate during the pandemic and children and adolescents used telemedicine the least. Telemedicine usage varied among patients with different chronic diseases, those with mental illness and with congestive heart failure making maximum use of telemedicine, and those with other ambulatory sensitive chronic conditions making substantial use of telemedicine. This study is the first to quantify the rates of telemedicine use in rural populations, particularly among older patients and those with chronic diseases.

Health care access has been a major challenge for rural patients in many jurisdictions, particularly for chronic disease management and access to specialist care. Previous studies have described the increasing trend of telemedicine use specifically among rural beneficiaries even prior to the pandemic [13]. Numerous advantages are associated with the adoption of telemedicine in rural regions, such as saving of time and costs associated with traveling, reduction of hospital readmissions, prevention of emergency department visits, and increasing the availability of inpatient beds for patients requiring critical care [14]. Rural telemedicine has been shown to improve health care access and quality in various areas, ranging from trauma and critical care [15-17], family medicine [18], pediatrics [19], and cancer care [20]. In fact, several randomized controlled trials have reported that telemedicine-based care can lead to similar and potentially better outcomes compared to in-person care among rural patients [21,22].

Furthermore, previous studies have reported that populations overall have increased their use of telemedicine and virtual care during the pandemic [23] to avail of both urgent and nonurgent care [24], and benefits have been observed within various subspecialties [25,26]. Telemedicine allows health care professionals to safely deliver care by reducing the risk of potential exposure to the care provider and the patient, preserving personal protective equipment, and lessening patient loads at hospitals and facilities [27]. This proves especially useful in rural settings, where a significant proportion of the population are older adults and are at a higher risk of COVID-19.

Telemedicine uptake has surged across several groups of patients with chronic diseases, potentially highlighting the importance of remote management of chronic conditions. This finding is concurrent with published data on the use of telemedicine among patients with various chronic diseases, such as diabetes [28] and congestive heart failure [29], and particularly among patients residing in rural or remote areas [30]. Although rates of telemedicine use increased among patients with chronic disease, the proportion of telemedicine users with chronic disease appeared to decrease slightly over the years, suggesting a potentially increased uptake of telemedicine overall. We hypothesize that with the widespread use of telemedicine, a greater proportion of healthier patients used telemedicine for their health care needs. Longitudinal studies on the changes in the trends of telemedicine usage, particularly in the postpandemic period, are needed.

Despite evidence on the effectiveness of virtual ambulatory care for older adults [31], some studies have reported the challenges of adopting telemedicine in this population, owing to barriers such as disabilities or lack of devices, proper internet connectivity, and experience with technology [32,33]. However, our study shows that telemedicine usage increased significantly among older adult patients during the pandemic. This was likely a result of the new COVID-19 billing codes, which allowed for the reimbursement of telephone visits, as telephone calls may be easier to access for older adults than videoconferencing. Our findings are concurrent with those of a similar study we conducted, which focused on the rates of telemedicine visits in the entire patient population in Ontario, and we previously reported that the increase in telemedicine usage rates from before the pandemic to during the pandemic was similar across age groups, with older adults showing the highest rates of telemedicine use during the pandemic [3].

### Limitations

The limitations of this study include a shortage of data comparing different modalities of communication technology, specifically telephone versus video visits. The new COVID-19 billing codes reimburse for telemedicine delivered through various modalities, and the type of modality used is not available in administrative data sets. The lack of clinical granularity that accompanies the use of administrative data also implies our inability to deduce the reasons for telemedicine use and assess the quality of care in our study population.

### Conclusions

Our study investigates the trend in telemedicine use among patients residing in rural Ontario before and during the COVID-19 pandemic. Rural patients globally face many barriers to care, and telemedicine has proved important in helping patients access the health care services they need. Uptake of telemedicine services increased after the onset of the pandemic for the rural patient population of Ontario and across various subgroups, including those who are older or those with chronic disease. Telemedicine use appears to have increased more significantly among patients residing in less rural areas compared to those residing in more rural areas during the pandemic. Further studies are required to assess the potential barriers to telemedicine experienced by rural populations compared to those experienced by urban populations and the impact of telemedicine compared to that of in-person care on other forms of health care utilization, outcomes, and quality of care among vulnerable and at-risk patient groups in the rural population.

## Appendix

Multimedia Appendix 1 Supplemental Tables.

Multimedia Appendix 2 Rate of telemedicine visits per 1000 rural patients, by region.

Multimedia Appendix 3 Medical specialties of physicians who delivered any rural visit in 2012, 2016, and 2020.

## Abbreviations

ICES: Institute for Clinical Evaluative Sciences
OHIP: Ontario Health Insurance Plan
OTN: Ontario Telemedicine Network
RIO: rurality index of Ontario
