# Supplementary material for: Rural Telemedicine Use Before and During the COVID-19 Pandemic: Repeated Cross-sectional Study
Source: J Med Internet Res. 2021 Apr 5;23(4):e26960. doi: 10.2196/26960 (PMC8023379; doi:10.2196/26960)
Supplement: Multimedia Appendix 1 [file jmir_v23i4e26960_app1.docx]

**Supplemental Table 1. Billing Codes for Telemedicine**

| Time Period | Billing Codes | Modality |
| --- | --- | --- |
| January 1, 2012- June 30, 2020 | B100, B200, B099 | Video-visit |
| April 1, 2020- June 30, 2020 | B103, B203, B209 | Video-visit |
| March 14, 2020- June 30, 2020 | K080, K081, K082, K083, H409, H410 | All telemedicine modalities |

**Supplemental Table 2. Inclusion Criteria for Chronic Disease Patient Cohorts**

| Chronic Disease | Inclusion Criteria |
| --- | --- |
| Serious mental illness | Patients with **serious mental illness** that affects functioning, characterized by at least 2 outpatient or 1 inpatient claims with the corresponding ICD 9 or 10 codes or DXCODES within 12 months prior to year of interest [NACRS, OHIP, DAD]:  Schizophrenia and psychotic disorders:  DAD/NACRS:  ICD-9=295, 297 ICD-10= F20.0, F20.1, F20.2, F20.5, F20.9, F20.81, F20.89, F22, F23, F24, F25.9  OHIP DXCODES: 295, 297, 298  Bipolar disorder:  DAD/NACRS: ICD-9=296.0, 296.1, 296.4-296.6, 296.7, 296.8, 296.9, 301.11, 301.13 ICD-10=F30.1, F30.2, F30.3, F30.4, F30.8, F31.1, F31.2, F31.3, F31.4, F31.5, F31.6, F31.73, F31.74, F31.75, F31.76, F31.77, F31.78, F31.81, F31.9, F32.8, F34.0, F34.8, F39, F60.89  OHIP DXCODES: 296 |
| COPD | Record in the ICES COPD database any time prior to year of interest |
| Heart failure | Record in ICES CHF database any time prior to year of interest |
| Asthma | Record in ICES ASTHMA database any time prior to year of interest |
| Hypertension | Record in the ICES HYPER database any time prior to year of interest |
| Angina | At least one ED visit within 12 months prior to year of interest with any of the following codes [NACRS, OHIP]:  ICD-9: 411.1, 413.0, 413.1, 413.9, 786.51, 786.52  ICD-10: R07.1–R07.4, I20.0, I20.1, I20.8, I20.88, or I20.9 |
| Diabetes | Record in the ICES ODD database any time prior to year of interest |
