# Supplementary figures and images for: Rural Telemedicine Use Before and During the COVID-19 Pandemic: Repeated Cross-sectional Study
Source: J Med Internet Res. 2021 Apr 5;23(4):e26960. doi: 10.2196/26960 (PMC8023379; doi:10.2196/26960)

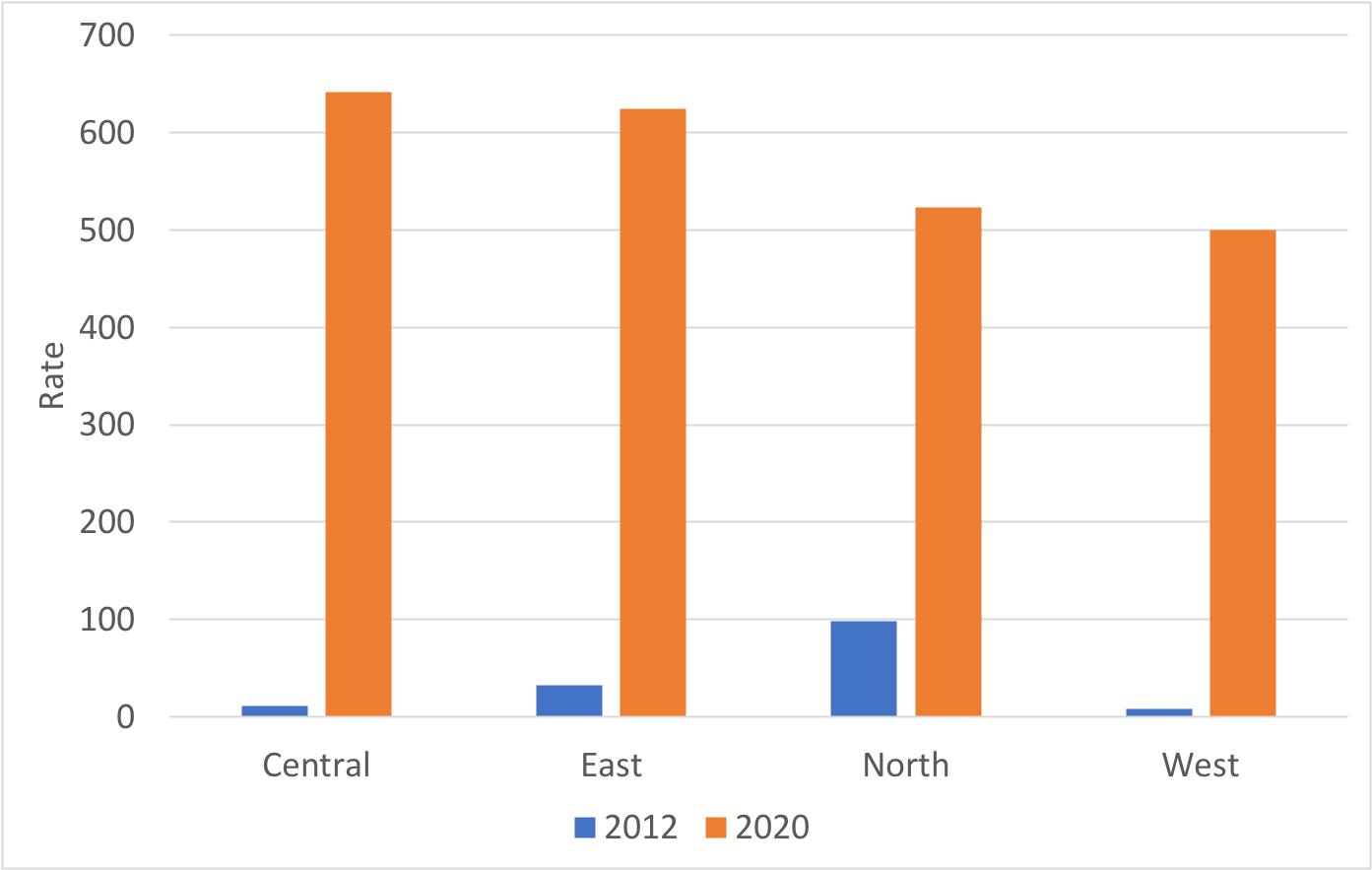

Supplement: Multimedia Appendix 2 [file jmir_v23i4e26960_app2.png]
