# Supplementary material for: Rural Telemedicine Use Before and During the COVID-19 Pandemic: Repeated Cross-sectional Study
Source: J Med Internet Res. 2021 Apr 5;23(4):e26960. doi: 10.2196/26960 (PMC8023379; doi:10.2196/26960)
Supplement: Multimedia Appendix 3 [file jmir_v23i4e26960_app3.docx]

**Appendix 2. Medical specialties of physicians who delivered any rural visit in 2012, 2016, and 2020**

| Medical Specialty | 2012 | | 2016 | | 2020 | |
| --- | --- | --- | --- | --- | --- | --- |
|  | **Zero TM visits**  **(N=21,699)** | **≥1 TM visits**  **(N=597)** | **Zero TM visits**  **(N=23,858)** | **≥1 TM visits**  **(N=1330)** | **Zero TM visits**  **(N=6,287)** | **≥1 TM visits**  **(N=17,601)** |
| Family/General Practice | 10,672 (49.2%) | 91 (15.2%) | 11,922 (50.0%) | 294 (22.1%) | 2,543 (40.4%) | 9044 (51.4%) |
| Internal Medicine | 1,238 (5.7%) | 51 (8.5%) | 1,191 (5.0%) | 74 (5.6%) | 453 (7.2%) | 656 (3.7%) |
| Pediatrics | 911 (4.2%) | 16 (2.7%) | 1,081 (4.5%) | 28 (2.1%) | 320 (5.1%) | 679 (3.9%) |
| Psychiatry | 1,299 (6.0%) | 101 (16.9%) | 1,255 (5.3%) | 218 (16.4%) | 253 (4.0%) | 1121 (6.4%) |
| Surgery | 1,615 (7.4%) | 72 (12.1%) | 1,676 (7.0%) | 155 (11.7%) | 470 (7.5%) | 1271 (7.2%) |
| Other | 5,964 (27.5%) | 266 (44.6%) | 6,733 (28.2%) | 561 (42.2%) | 2,248 (35.8%) | 4830 (27.4%) |

**TM = telemedicine*
